# Cytokine profiling of molecular endotypes of knee osteoarthritis: insights from the IMI-APPROACH cohort

**DOI:** 10.1186/s13075-026-03742-9

**Published:** 2026-01-27

**Authors:** Monica T. Hannani, Jaume Bacardit, Jonathan Larkin, Virpi Glumoff, Morten A. Karsdal, Anne-Christine Bay-Jensen, Ali Mobasheri, Christian S. Thudium

**Affiliations:** 1 Nordic Bioscience A/S, Herlev Hovedgade 205-207, Herlev, 2730 Denmark; 2 Department of Biomedical Sciences, Faculty of Health and Medical Sciences, University of Copenhagen, Copenhagen, Denmark; 3 Interdisciplinary Computing and Complex BioSystems (ICOS) research group, School of Computing, Newcastle University, Newcastle upon Tyne, UK; 4 Research Unit of Health Sciences and Technology, Faculty of Medicine, University of Oulu, Oulu, Finland; 5 SynOA Therapeutics, Philadelphia, PA USA; 6 Medical Research Laboratory Unit, Faculty of Medicine, University of Oulu, Oulu, Finland; 7 Department of Regenerative Medicine, State Research Institute Centre for Innovative Medicine, Vilnius, Lithuania; 8 World Health Organization Collaborating Centre for Public Health Aspects of Musculoskeletal Health and Aging, University of Liège, Liège, Belgium; 9 Department of Joint Surgery, First Affiliated Hospital of Sun Yat-sen University, Guangzhou, China

**Keywords:** Biomarker, Cytokine, Endotype, Endotyping, IL-1ra, Inflammation, Osteoarthritis

## Abstract

**Background:**

Molecular endotypes that decipher the heterogeneity of osteoarthritis (OA) have been described. This exploratory study aimed to further the molecular understanding of three previously identified biomarker-based endotypes of knee OA through cytokine profiling.

**Methods:**

Fifteen pro- and anti-inflammatory cytokines were measured in serum at the six-, 12-, and 24-month follow-up visits of 277 knee OA participants from IMI-APPROACH using Luminex multiplexed immunoassays. Longitudinal differences in cytokine levels between previously defined endotype subgroups; (i) structural damage to bone and cartilage, (ii) low tissue turnover, and (iii) connective tissue inflammation were estimated with linear mixed-effects models, adjusting for patient-specific random effects, age, sex, and BMI. Within-patient stability of the cytokines over 18 months was compared to 19 tissue turnover biomarkers of which defined the endotypes.

**Results:**

Compared to tissue-turnover biomarkers measured in IMI-APPROACH, the panel of 15 cytokines demonstrated increased fluctuations over time with lower within-patient stability and less discriminatory abilities between the endotype. Only the anti-inflammatory cytokine interleukin-1 receptor antagonist (IL-1ra) was consistently and differentially elevated in the inflammatory endotype subgroup (*n* = 92). At the 12-month visit, a mean change of IL-1ra of 36% (95% CI: 19%, 54%; p-value < 0.001) was found for the inflammatory endotype relative to the structural damage endotype, and 40% (95% CI: 22%, 59%; *p*-value < 0.001) at month 24. At the 24-month visit, a mean change of IL-1ra of 21% (95% CI: 10%, 31%; p-value = 0.047) was found for the inflammatory endotype relative to the low tissue turnover endotype. Participants with the highest quartile expression of IL-1ra within the inflammatory endotype (*n* = 23) exhibited higher BMI (*p* = 0.035, Mann-Whitney U test) and worsened Western Ontario and McMaster Universities Osteoarthritis Index (WOMAC) function (*p* = 0.035, Mann-Whitney U test) compared to the lowest quartile of IL-1ra expression.

**Conclusions:**

This study found that the majority of the cytokines exhibited considerable fluctuations over time with no endotype-specific cytokine profiles. This study indicates that while the included cytokines are important for the understanding of OA pathology, they may not be stable reflections of the endotypic profiles of KOA over time.

**Supplementary Information:**

The online version contains supplementary material available at 10.1186/s13075-026-03742-9.

## Background

The incomplete understanding of the heterogeneous subpopulations of osteoarthritis (OA) has hampered the successful development of disease-modifying treatment options. To address the heterogeneity of OA and uncover the pathobiological pathways that drive the disease, the interest in molecular endotyping has surged in recent years [1]. An endotype is classified as a disease subpopulation that is mechanistically defined by distinct pathways that drive the disease. Endotyping may therefore provide a more precise definition of patient subgroups than observable phenotypes and holds the potential of informing targeted treatment-strategies for OA patients in the future [2]. Numerous endotypes have been described by the OA community in recent years. The Innovative Medicines Initiative Applied Public-Private Research enabling Osteoarthritis Clinical Headway (IMI-APPROACH) consortium presented three molecular endotypes in a two-year European observational cohort of 295 knee OA (KOA) participants [3]. These were identified through *k*-means clustering of 16 biochemical markers of tissue turnover. The endotype driven by structural damage to bone and cartilage was defined by high levels of bone (sCTX-I, u-αCTX-I, and N-MID) and cartilage (uCTX-II) turnover biomarkers. The inflammatory endotype was defined by high levels of systemic (high-sensitivity C-reactive protein [hsCRP]) and connective tissue (CRPM and C3M) inflammation biomarkers. The low tissue turnover endotype was defined by low levels of most biomarkers [3]. It has recently been shown that more than half of the participants’ endotypes remained stable over 18 months, which is an important aspect for the clinical applicability and treatability of molecular endotyping [4]. However, a better understanding of these endotypes are needed and their potential clinical utility has not yet been established.

While several molecular endotypes of OA have been presented by the field, the inflammatory endotype is arguably the most well-described to date and has garnered increasing attention due to its potential for targeted treatments [5, 6]. Inflammatory OA endotypes such as endotypes driven by synovial inflammation [7], inflammation-associated senescence [8], and obesity-related inflammation [9] have been described but a comprehensive molecular understanding of the inflammatory underpinnings of OA endotypes remains uncovered. Many pathophysiological processes leading to joint destruction in OA are driven by inflammatory mediators including cytokines and chemokines. Some of the most important pro-inflammatory mediators of OA include interleukin (IL)-1β, tumor necrosis factor (TNF)-α, IL-6, IL-15, IL-17, and IL-18. Anti-inflammatory cytokines are also important actors in OA by modulating the inflammatory response to protect the affected joints. Some of the most well-described anti-inflammatory cytokines in OA include IL-4, IL-10, and IL-13 [10]. However, how such pro- and anti-inflammatory cytokines can be associated with and used to accurately describe the presented endotypes of OA is not well understood.

In light of ongoing treatment studies targeting inflammatory components of OA, including intra-articular administration of a glucagon-like peptide-1 analog (liraglutide) [11] and gene therapy of recombinant IL-1 receptor antagonist (IL-1ra) [12], a better characterization of the inflammatory elements of OA endotypes is urgently needed [5]. In doing so, it may be possible to correctly identify OA patients with endotypes that will show benefit from disease-modifying OA treatments, such as anti-inflammatory treatment options, for future drug development.

The aim of this exploratory study was to perform pro- and anti-inflammatory cytokine-profiling to further the molecular understanding of the structural damage, low tissue turnover, and inflammatory endotypes originally described in IMI-APPROACH [3, 4] and to investigate whether the cytokines are specific for the inflammatory endotype or endotype-independent. This study also explores the longitudinal stability of the cytokines and their applicability as biomarkers for such endotypes.

## Methods

### Aim

The aim of this exploratory study was to perform cytokine-profiling of three biomarker-based endotypes of KOA and to assess the longitudinal stability of the cytokines and their applicability for molecular endotyping of KOA.

### Participants

295 participants fulfilling the American College of Rheumatology criteria for tibiofemoral KOA from the European, multi-center, observational IMI-APPROCH cohort (ClinicalTrials.gov ID: NCT03883568) were considered. IMI-APPROACH was a unique cohort in which KOA participants (Kellgren-Lawrence [KL] grades 0–4) were recruited from existing observational cohorts based on a higher likelihood of structural and/or pain progression over two-years using machine learning models trained on the CHECK cohort [13, 14]. Van Helvoort et al. (2020) is referred to for an in-depth cohort description [14]. KOA participants assigned to the biomarker-based endotype subgroups (i) structural damage, (ii) inflammation, or (iii) low tissue turnover with data available at the six- (*n* = 277), 12- (*n* = 261), and 24-month (*n* = 234) visits were included in this study [4]. Of the KOA participants with data available at all visits (*n* = 226), 123 exhibited a longitudinally stable endotype (defined as being independently assigned to the same biomarker-based endotype at all visits).

### Cytokine and biochemical marker data

The Human Immunotherapy Luminex^®^ Performance Multiplex platform (R&D Systems Inc., USA) quantifies 24 pro- and anti-inflammatory cytokines that are associated with host response to immune-based therapies. The cytokine panel was measured at all visits in serum according to the manufacturer’s protocol. Longitudinal measurements of 19 serum (s) and urine (u) biomarkers used to define the endotypes [3] were included as described in Hannani et al. (2025) [4]. The biomarkers reflected bone turnover (sCTX-I, N-MID, PRO-C1, and u-αCTX-I), cartilage turnover (ARGS-aggrecan, C2M, C10C, cartilage oligomeric matrix protein [COMP], Coll2–1, Coll2–1NO_2_, hyaluronic acid [HA], PRO-C2, and uCTX-II), and inflammation (C1M, C3M, CRPM, hsCRP, PRO-C4, and VICM) [2].

### Data preprocessing

Nine cytokines with > 50% missing data due to being below the detection limit at the first visit were excluded (CD40L, Granzyme B, interferon [IFN]-α, IFN-γ, IL-4, IL-6, IL-13, IL-33, and PD-L1), resulting in 15 cytokines included for downstream analyses (granulocyte-macrophage colony-stimulating factor [GM-CSF], IL-1α, IL-1β, IL-1ra, IL-2, IL-8, IL-10, IL-12p70, IL-15, IL-17 A, IFN-γ-induced protein [IP]-10, monocyte chemoattractant protein [MCP]-1, macrophage inflammatory protein [MIP]-1α, MIP-1β, and TNF-α) (Supplementary Table 1). Cytokine (*n* = 15) and biomarker (*n* = 19) levels were natural logarithm-transformed and winsorized with Tukey’s rule [3]. For each cytokine and visit (month six, 12, and 24), measurements above Q3 + 1.5 x interquartile range (IQR) were replaced with the 98th quantile and measurements below the Q1–1.5 x IQR were replaced with the 2nd quantile to limit the effect of extreme outliers [3].

### Cytokine profiling and *k*-means clustering

For visualization of cytokine profiles, sex-specific z-score scaling was performed for each visit and cytokine [4]. Cytokine profiles of KOA endotype subgroups (the total population and the subset with longitudinally stable endotypes) were visualized as radar charts for each visit. Min-max normalized median z-score scaled concentrations (rescaling of median values to range between 0 and 1 using minimum and maximum median values) were utilized.

*K*-means clustering of the natural log-transformed levels of 15 cytokine was performed at the six-month visit, as described in Hannani et al. (2025) [4]. For the clustering procedure only, missing data (14.4%) was imputed with a random forest model using the *missForest* package with ntree = 300. Sex-specific z-score scaling was performed on the imputed cytokine data, and a principal component analysis was performed to reduce the effect of correlations between the cytokines. The principal components that explained 95% of the variance were used as input for the *kmeans* algorithm using nstart = 35 and iter.max = 25. Cytokine profiles of the three cytokine-based clusters were visualized as radar charts, as previously described.

### Intraclass correlation coefficients of cytokines and biomarkers

Intraclass correlation coefficients (ICCs) and their 95% confidence intervals (CIs) were estimated of natural logarithm-transformed non-scaled cytokine and biomarker levels over time (month six, 12, and 24). A two-way mixed-effects model was used, considering the average of three ratings (ICC3K model) [15] with *ICC* from the *psych* package.

### Cytokine expression differences between endotypes

Percent changes in geometric mean cytokine concentrations between the endotype subgroups were estimated with linear mixed-effects models (LMMs). The LMMs were run with an interaction term between endotypes and visit and were adjusted for participant-specific random effects. The changes were estimated with and without adjusting for age, sex, and body mass index (BMI). Changes were estimated using natural logarithm-transformed non-scaled cytokine levels (Supplemental File 1).

### Statistical analysis

All data analysis was performed using R version 4.4.2. Differences in clinical characteristics were assessed with Pearson’s χ^2^ tests for categorical variables and Mann-Whitney U tests for numerical variables. All p-values were Benjamini-Hochberg-adjusted for multiple comparisons.

## Results

### Correlation of cytokines and biomarkers with comorbidities

Cytokine profiling of 24 pro- and anti-inflammatory cytokines was performed in serum of KOA participants from the observational IMI-APPROACH cohort with molecular endotypes of (i) structural damage to bone and cartilage, (ii) low tissue turnover, or (iii) connective tissue inflammation, discovered through *k*-means clustering of tissue turnover biomarkers by Angelini et al. (2022) [3]. For this study, longitudinal biochemical data and endotype information from the follow-up visits after six (*n* = 277), 12 (*n* = 261), and 24 (*n* = 234) months were considered [4]. 78% (215/277) of the participants were female with a mean age of 66.6 ± 7.0 years and mean BMI of 28.0 ± 5.3 (Table 1). Of reported comorbidities, hypertension was the most common with a frequency of 33.2% (92/277) with no differences in proportion of hypertensive participants between the endotype subgroups. Of the 24 cytokines quantified with the Luminex^®^ multiplex platform, nine cytokines had over 50% of measurements below the detection limit at the first visit and were excluded from the study. No endotype-specific patterns of missingness were observed for the cytokines across all visits (Supplementary Fig. 1). As a result, the 15 cytokines included in this study were GM-CSF, IL-1α, IL-1β, IL-1ra, IL-2, IL-8, IL-10, IL-12p70, IL-15, IL-17A, IP-10, MCP-1, MIP-1α, MIP-1β, and TNF-α.

**Table 1:** Clinical characteristics of endotypes

	Inflammatory (*n* = 92)^a^	Structural damage (*n* = 104)^b^	Low tissue turnover (*n* = 81)^c^	All (*n* = 277)^d^
Sex				
Female	73 (79.3%)	82 (78.8%)	60 (74.1%)	215 (77.6%)
Age (years)				
Mean (SD)	66.35 (6.91)	66.87 (6.54)	66.47 (7.71)	66.58 (7.00)
BMI (kg/m^2^)				
Mean (SD)	29.50 (5.63)	26.21 (4.44)	28.73 (5.29)	28.04 (5.29)
KL grade				
0	13 (14.8%)	19 (18.6%)	15 (19.7%)	47 (17.7%)
1	22 (25.0%)	26 (25.5%)	20 (26.3%)	68 (25.6%)
2	24 (27.3%)	20 (19.6%)	16 (21.1%)	60 (22.6%)
3	28 (31.8%)	34 (33.3%)	19 (25.0%)	81 (30.5%)
4	1 (1.1%)	3 (2.9%)	6 (7.9%)	10 (3.8%)
JSW medial (mm)				
Mean (SD)	4.18 (1.24)	4.25 (1.26)	4.33 (1.29)	4.25 (1.26)
JSW lateral (mm)				
Mean (SD)	7.12 (1.62)	6.69 (1.65)	7.01 (1.57)	6.92 (1.62)
JSW min (mm)				
Mean (SD)	2.42 (1.12)	2.39 (1.19)	2.63 (1.17)	2.47 (1.16)
WOMAC Pain (%)				
Mean (SD)	31.69 (21.17)	27.74 (20.63)	30.25 (23.45)	29.76 (21.64)
WOMAC Function (%)				
Mean (SD)	33.32 (21.25)	26.90 (20.08)	31.25 (22.39)	30.30 (21.27)
WOMAC Stiffness (%)				
Mean (SD)	41.62 (25.00)	33.74 (24.31)	35.47 (26.64)	36.86 (25.38)
Comorbidities				
Asthma	17 (18.5%)	13 (12.5%)	9 (11.1%)	39 (14.1%)
Cancer	6 (6.5%)	13 (12.5%)	10 (12.3%)	29 (10.5%)
Cerebrovascular disease	5 (5.4%)	3 (2.9%)	5 (6.2%)	13 (4.7%)
Congestive heart failure	1 (1.1%)	1 (1.0%)	1 (1.2%)	3 (1.1%)
Depression	12 (13.0%)	11 (10.6%)	10 (12.3%)	33 (11.9%)
Diabetes	6 (6.5%)	2 (1.9%)	7 (8.6%)	15 (5.4%)
Gastric peptic ulcer	2 (2.2%)	8 (7.7%)	1 (1.2%)	11 (4.0%)
Hemiplegia	1 (1.1%)	0 (0.0%)	1 (1.2%)	2 (0.7%)
Hypertension	31 (33.7%)	32 (30.8%)	29 (35.8%)	92 (33.2%)
Liver disease	3 (3.3%)	5 (4.8%)	2 (2.5%)	10 (3.6%)
Myocardial infarct	2 (2.2%)	3 (2.9%)	3 (3.7%)	8 (2.9%)
Perivascular disease	3 (3.3%)	5 (4.8%)	4 (4.9%)	12 (4.3%)
Renal disease	2 (2.2%)	2 (1.9%)	1 (1.2%)	5 (1.8%)
Rheumatic disease	4 (4.3%)	2 (1.9%)	2 (2.5%)	8 (2.9%)
Skin ulcers	3 (3.3%)	5 (4.8%)	3 (3.7%)	11 (4.0%)

Target knee data from the first visit (month six) was considered (*n* = 277). Higher Western Ontario and McMaster Universities Osteoarthritis Index (WOMAC) scores indicate worse symptoms. KL, Kellgren-Lawrence; JSW, joint-space width; SD, Standard deviation

^a^Missing data: BMI (*n* = 1), KL grade (*n* = 4), JSW medial (*n* = 5), JSW lateral (*n* = 5), JSW min (*n* = 5), WOMAC Pain (*n* = 3), WOMAC Function (*n* = 7), WOMAC Stiffness (*n* = 1)

^b^Missing data: KL grade (*n* = 2), JSW medial (*n* = 3), JSW lateral (*n* = 1), JSW min (*n* = 1), WOMAC Function (*n* = 7), WOMAC Stiffness (*n* = 1)

^c^Missing data: KL grade (*n* = 5), JSW medial (*n* = 3), JSW lateral (*n* = 2), JSW min (*n* = 2), WOMAC Pain (*n* = 2), WOMAC Function (*n* = 4), WOMAC Stiffness (*n* = 1)

^d^Missing data: BMI (*n* = 1), KL grade (*n* = 11), JSW medial (*n* = 11), JSW lateral (*n* = 8), JSW min (*n* = 8), WOMAC Pain (*n* = 5), WOMAC Function (*n* = 18), WOMAC Stiffness (*n* = 3)

**Fig. 1:**
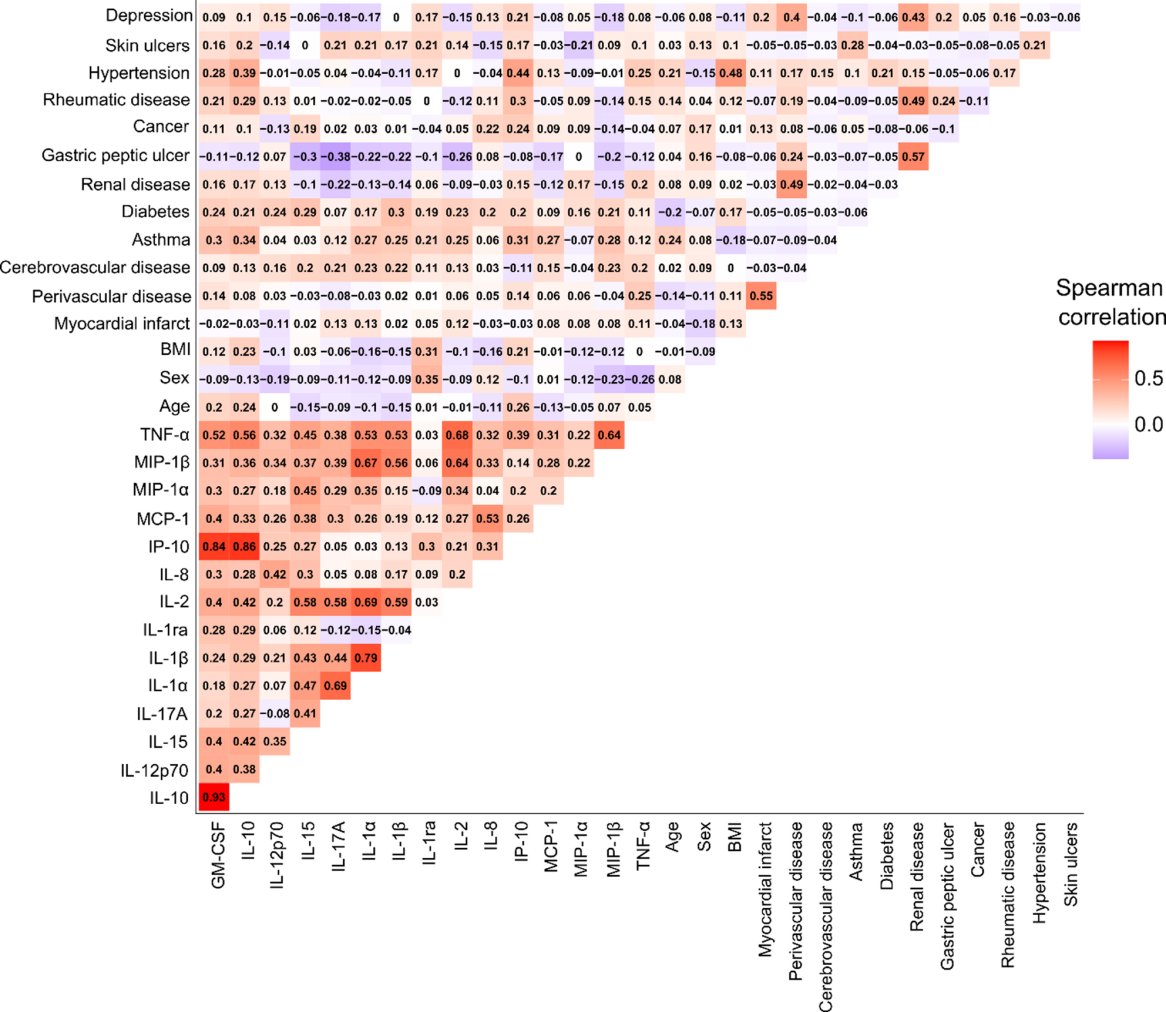
Spearman’s correlations between cytokines, clinical characteristics, and reported comorbidities at the six-month visit

The highest Spearman’s correlation between the cytokines, clinical characteristics (age, sex, and BMI), and reported comorbidities at the six-month visit were observed for hypertension and IP-10 (*ρ* = 0.44) as well as IL-10 (*ρ* = 0.39) (Fig. 1), with both cytokines having previously been associated with hypertension [16, 17]. Of the biomarkers originally used to define the endotype subgroups [3], the highest Spearman’s correlations with clinical characteristics were found for hsCRP and BMI (*ρ* = 0.42) and u-αCTX-I and sex (*ρ* = 0.33), with weak correlations to the reported comorbidities at the six-month visit (Supplementary Fig. 2).

### Unstable cytokine expression patterns over time

**Fig. 2:**
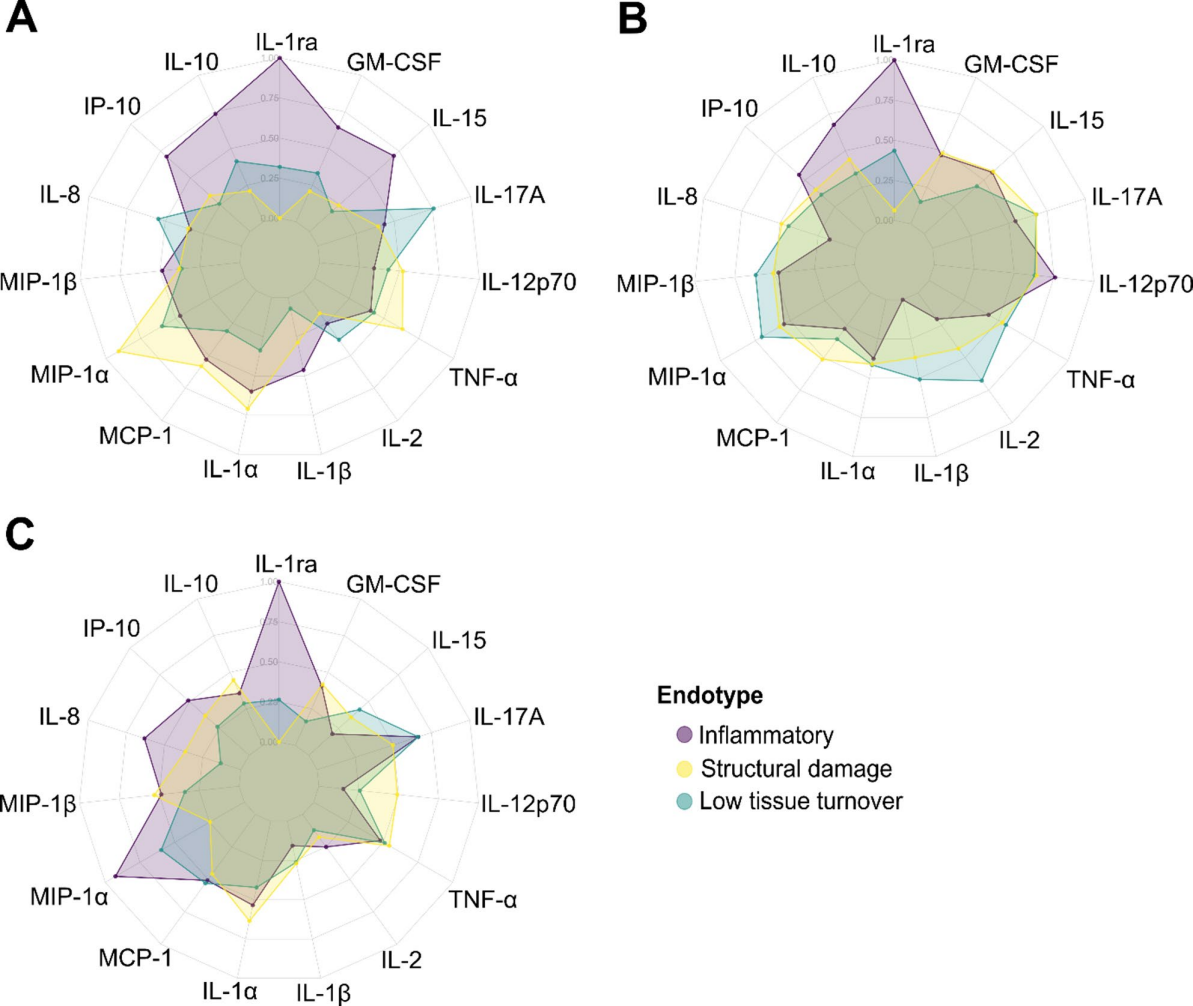
Cytokine profiles of endotypes at the (**A**) six-, (**B**) 12-, and (**C**) 24-month visits

**Fig. 3:**
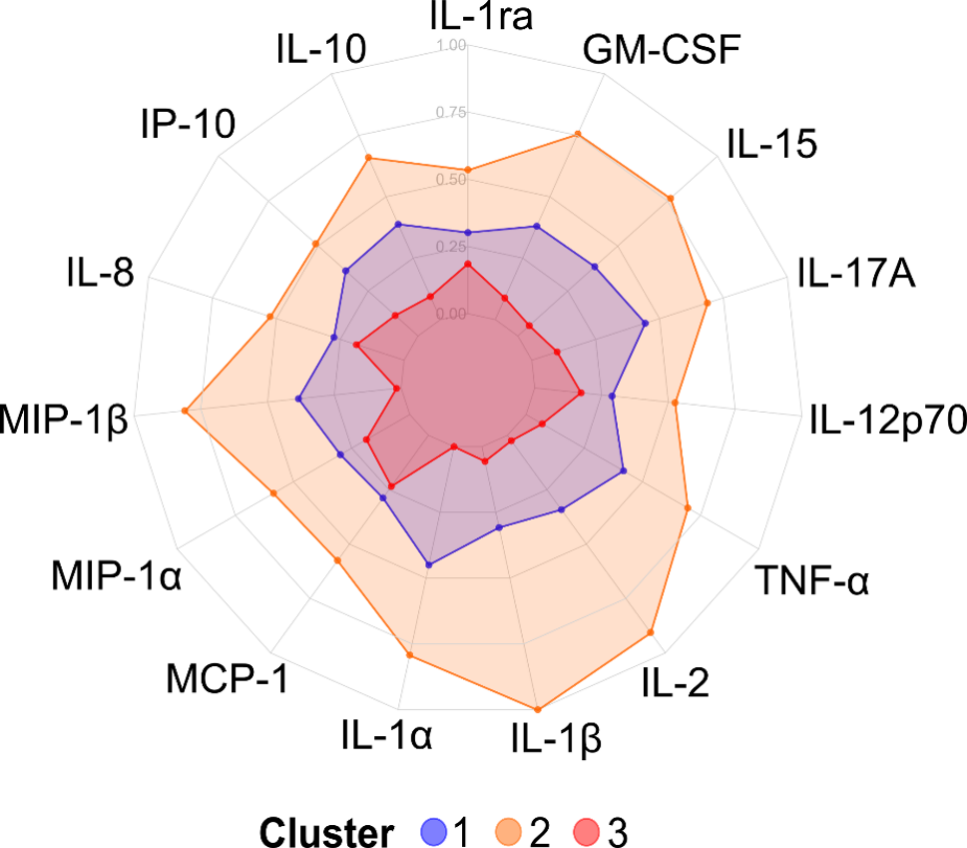
Cytokine profiles of three clusters obtained by *k*-means clustering of sex-specific z-score scaled cytokine levels

Cytokine profiling of the molecular endotype subgroups revealed fluctuating cytokine expression patterns over three visits, spanning 18 months (Fig. 2A-C). For participants with a longitudinally stable endotype (independently assigned to the same endotype at all visits) (*n* = 123) [4], fluctuating cytokine profiles were observed as well (Supplementary Fig. 3). To investigate whether the 15 cytokines could provide meaningful segregation of the KOA participants, *k-*means clustering was performed on the cytokine data as was originally done with the tissue turnover biomarkers to define the endotype subgroups [3, 4]. No meaningful clusters were obtained. Rather, the KOA participants were generally separated into overall high, middle, and low cytokine expression groups with no distinct expression patterns between the groups (Fig. 3 & Supplementary Fig. 4).

### Increased IL-1ra expression in the inflammatory endotype

**Fig. 4:**
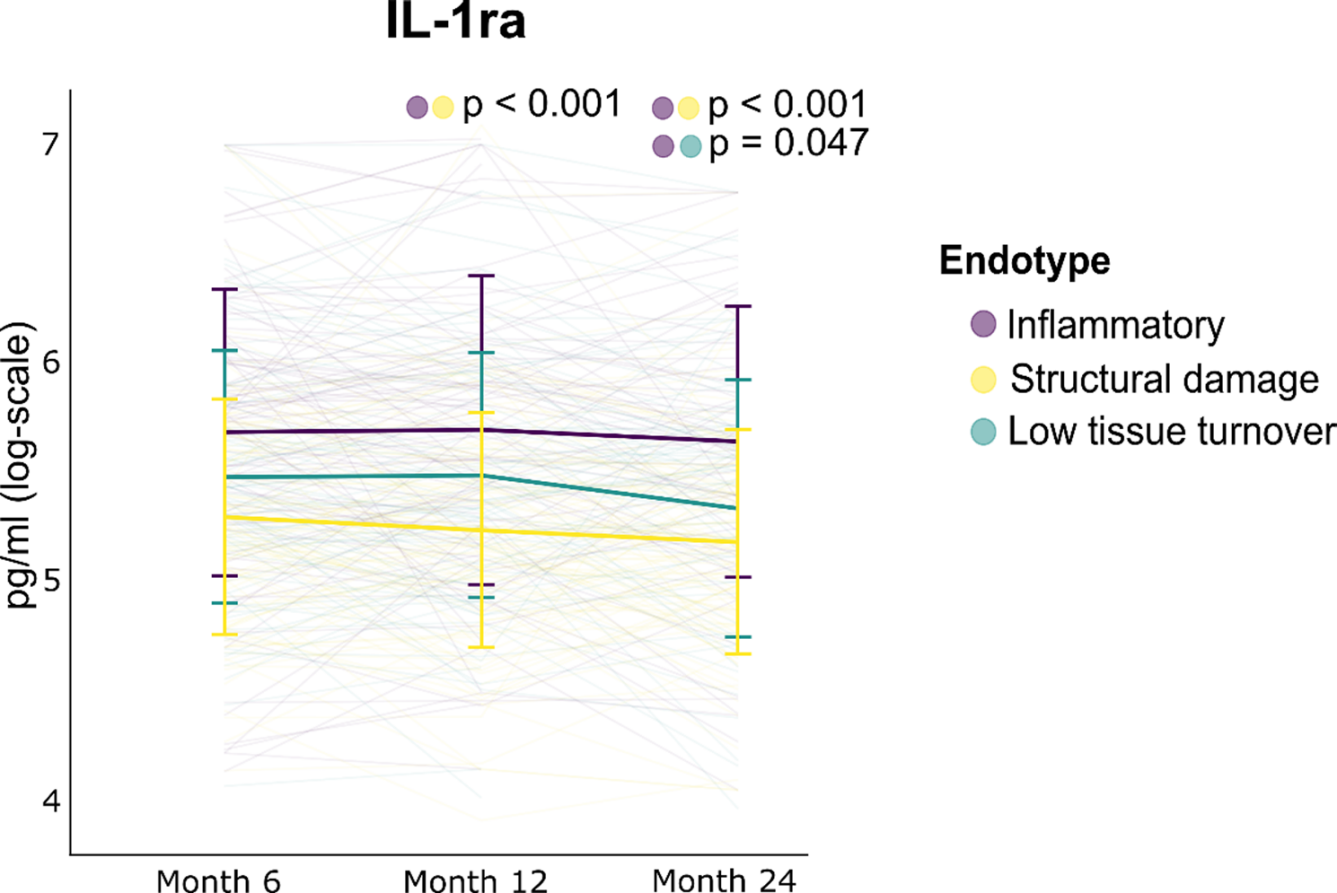
Longitudinal natural logarithm-transformed concentrations of interleukin-1 receptor antagonist (IL-1ra) across endotypes, showing Benjamini-Hochberg-adjusted *p*-values

IL-1ra was the only cytokine to consistently exhibit differences between the endotype subgroups, when adjusting for confounders (age, sex, and BMI) (Fig. 4). On the 12-month visit, a mean change of IL-1ra of 36% (95% CI: 19%, 54%; p-value < 0.001) was found for the inflammatory endotype subgroup relative to the structural damage endotype, while it was 40% (95% CI: 22%, 59%; p-value < 0.001) by month 24 (Supplementary Table 2). On the 24-month visit, a mean change of IL-1ra of 21% (95% CI: 10%, 31%; p-value = 0.047) was found for the inflammatory endotype subgroup relative to the low tissue turnover endotype. Considering the clinical differences between KOA participants within the inflammatory endotype subgroup with the lowest (*n* = 23) and highest (*n* = 23) quartile expression of IL-1ra at the first visit, the highest IL-1ra quartile subgroup had higher BMI (p-value = 0.035, Mann-Whitney U test) and worsening of Western Ontario and McMaster Universities Osteoarthritis Index (WOMAC) function (p-value = 0.035, Mann-Whitney U test) (Table 2). No differences were found in changes in the clinical characteristics from month six to 24 between the IL-1ra quartile subgroups (Supplementary Table 3). The longitudinal stability of the inflammatory endotype did not appear to be related to levels of IL-1ra as the same rates of participants with a stable inflammatory endotype were found in the lowest (*n* = 10/23) and highest (*n* = 11/23) IL-1ra quartile groups (Supplementary Table 4).

**Table 2:** Clinical characteristics of participants in lower and upper IL-1ra expression quartile within the inflammatory endotype

	Low IL-1ra inflammatory endotype (*n* = 23)^a^	High IL-1ra inflammatory endotype (*n* = 23)^b^	*p*-value	Adjusted *p*-value
Sex			0.460	0.527
Female	20 (87%)	17 (73.9%)		
Age (years)			0.461	0.527
Median (Q1, Q3)	66.0 (62.0, 69.5)	63.0 (59.0, 70.0)		
Mean (SD)	66.0 (6.3)	64.6 (6.5)		
BMI (kg/m^2^)			0.007	0.035
Median (Q1, Q3)	26.2 (23.7, 28.9)	32.6 (28.4, 36.0)		
Mean (SD)	27.1 (5.6)	32.0 (5.7)		
KL grade			0.215	0.431
0	1 (4.8%)	5 (23.8%)		
1	6 (28.6%)	4 (19.%)		
2	9 (42.9%)	5 (23.8%)		
3	5 (23.8%)	7 (33.3%)		
JSW medial (mm)			0.841	0.841
Median (Q1, Q3)	4.28 (3.48, 5.03)	4.31 (3.27, 4.69)		
Mean (SD)	4.24 (1.16)	4.03 (1.38)		
WOMAC Pain (%)			0.019	0.052
Median (Q1, Q3)	25.0 (15.0, 42.5)	50.0 (30.0, 63.8)		
Mean (SD)	27.6 (19.2)	43.6 (23.1)		
WOMAC Function (%)			0.009	0.035
Median (Q1, Q3)	29.4 (15.4, 49.3)	45.6 (35.3, 60.3)		
Mean (SD)	29.8 (18.4)	47.7 (21.3)		
WOMAC Stiffness (%)			0.376	0.527
Median (Q1, Q3)	37.5 (25.0, 62.5)	50.0 (25.0, 68.8)		
Mean (SD)	40.3 (24.1)	47.3 (27.2)		

Target knee data from the first visit (month six) was considered. Higher Western Ontario and McMaster Universities Osteoarthritis Index (WOMAC) scores indicate worse symptoms. Pearson’s χ^2^ test was used for categorical variables and Mann-Whitney U test was used for numerical variables. *P*-values were Benjamini-Hochberg-adjusted for multiple comparisons

*IL-1ra* Interleukin-1 receptor antagonist, *KL* Kellgren-Lawrence, *JSW* Joint-space width, *SD* Standard deviation

^a^Missing data: KL grade (*n* = 2), JSW medial (*n* = 2), WOMAC Stiffness (*n* = 1)

^b^Missing data: BMI (*n* = 1), KL grade (*n* = 2), JSW medial (*n* = 2), WOMAC Pain (*n* = 1), WOMAC Function (*n* = 4)

### Lower stability of cytokines compared to tissue turnover biomarkers

The within-subject stability of the cytokine and biomarker levels over three visits (spanning 18 months) were estimated as ICCs. 53% (8/15) of the cytokines exhibited moderate stability over time (95% CI of ICC estimated between 0.5 and 0.75) [15] and 40% (6/15) of the cytokines exhibited good stability over time (95% CI of ICC estimated between 0.75 and 0.9) (Table 3). The highest stability of the cytokines was achieved for MCP-1 with an ICC of 0.89 (95% CI; 0.87, 0.91) and IL-1ra with an ICC of 0.88 (95% CI; 0.85, 0.90). The cytokines with the lowest within-subject stability were IL-17 A (ICC 0.49, 95% CI; 0.38, 0.58) and IL-2 (ICC 0.57, 95% CI; 0.48, 0.65), with poor stability over time. In comparison, 53% (10/19) of the tissue turnover biomarkers achieved good stability over time and 32% (6/19) exhibited excellent stability over time. The most stable biochemical markers were the type I collagen-derived biomarkers C1M and PRO-C1 both with an ICC of 0.94 (95% CI; 0.92, 0.95). The tissue-turnover biomarker with the lowest within-subject stability was the urinary uCTX-II (ICC 0.44, 95% CI; 0.31, 0.54).

**Table 3:** Intraclass correlation coefficients of 15 cytokines and 19 tissue-turnover biomarkers across all visits

Marker	Marker type	ICC (95% CI)
IL-1ra	Anti-inflammatory cytokine	0.88 (0.85, 0.90)
IL-10	Anti-inflammatory cytokine	0.78 (0.73, 0.82)
IL-8	Chemokine	0.86 (0.83, 0.89)
IP-10	Chemokine	0.86 (0.83, 0.89)
MCP-1	Chemokine	0.89 (0.87, 0.91)
MIP-1α	Chemokine	0.64 (0.57, 0.71)
MIP-1β	Chemokine	0.77 (0.72, 0.82)
GM-CSF	Pro-inflammatory cytokine	0.82 (0.78, 0.86)
IL-1α	Pro-inflammatory cytokine	0.65 (0.57, 0.72)
IL-1β	Pro-inflammatory cytokine	0.60 (0.51, 0.68)
IL-2	Pro-inflammatory cytokine	0.57 (0.48, 0.65)
IL-12p70	Pro-inflammatory cytokine	0.60 (0.51, 0.68)
IL-15	Pro-inflammatory cytokine	0.66 (0.59, 0.73)
IL-17 A	Pro-inflammatory cytokine	0.49 (0.38, 0.58)
TNF-α	Pro-inflammatory cytokine	0.80 (0.75, 0.83)
sCTX-I	Bone turnover biomarker	0.82 (0.78, 0.86)
N-MID	Bone turnover biomarker	0.93 (0.92, 0.94)
PRO-C1	Bone turnover biomarker	0.94 (0.92, 0.95)
u-αCTX-I	Bone turnover biomarker	0.74 (0.68, 0.79)
ARGS-aggrecan	Cartilage turnover biomarker	0.93 (0.91, 0.94)
C2M	Cartilage turnover biomarker	0.92 (0.90, 0.93)
C10C	Cartilage turnover biomarker	0.90 (0.88, 0.92)
COMP	Cartilage turnover biomarker	0.80 (0.76, 0.84)
Coll2-1	Cartilage turnover biomarker	0.77 (0.72, 0.81)
Coll2-1NO_2_	Cartilage turnover biomarker	0.91 (0.89, 0.93)
HA	Cartilage turnover biomarker	0.83 (0.79, 0.86)
PRO-C2	Cartilage turnover biomarker	0.83 (0.80, 0.86)
uCTX-II	Cartilage turnover biomarker	0.44 (0.31, 0.54)
C1M	Inflammation biomarker	0.94 (0.92, 0.95)
C3M	Inflammation biomarker	0.89 (0.87, 0.91)
CRPM	Inflammation biomarker	0.91 (0.89, 0.92)
hsCRP	Inflammation biomarker	0.82 (0.78, 0.86)
PRO-C4	Inflammation biomarker	0.92 (0.90, 0.93)
VICM	Inflammation biomarker	0.82 (0.78, 0.85)

Urinary markers (u) have been corrected for urinary creatinine levels

*CI* Confidence interval, *ICC* Intraclass correlation coefficient

## Discussion

Molecular endotyping has paved the way for addressing the substantial heterogeneity of OA [1]. To treat the disease-driving elements of OA and create clinical value, an in-depth understanding of the molecular mechanisms driving the endotypes is pivotal.

The aim of this exploratory study was to perform cytokine profiling in 277 KOA participants from IMI-APPROACH [3] to provide a better understanding of the inflammatory elements of the endotype subgroups driven by structural damage, low tissue turnover, and inflammation through an independent modality from which they were discovered. Quantification of 15 pro- and anti-inflammatory cytokines across three visits (spanning approximately 18 months) was associated with longitudinal fluctuations with no distinct cytokine profiles that were specific to one endotype. Several cytokines exhibited poor stability over time, such as the pro-inflammatory cytokines IL-17 A and IL-2. Within-subject fluctuations of such cytokines could be due to acute inflammatory flares [18, 19] or other external factors. Surprisingly, there was no consistent enrichment of a pro-inflammatory cytokine profile of the inflammatory endotype and only IL-1ra was differentially associated with the inflammatory endotype over time. No pro-inflammatory cytokines, such as IL-1α, IL-1β, or tumor necrosis factor (TNF)-α, were significantly elevated in the inflammatory endotype in serum. This apparent contradiction may be explained by how the inflammatory endotype was defined. The inflammatory endotype was primarily characterized by biomarkers of local tissue inflammation (such as C3M and CRPM) [3]. Such biomarkers can be considered downstream reflections of inflammation and increased cytokine load over time that may not directly translate to high systemic cytokine levels.

Within the inflammatory endotype, individuals in the highest quartile of IL-1ra expression had higher BMI and worsening of WOMAC function compared to the lowest quartile. IL-1ra has previously been shown to both associate with BMI and to be a causal predictor of radiographic progression of joint-space narrowing [20]. While it may appear curious that the highest expression of the anti-inflammatory cytokine was found for the inflammatory endotype, IL-1ra is induced by numerous inflammatory mediators such as IL-1, IL-4, and IFN-γ, and is therefore considered a downstream response to inflammation within the joint [20]. The inflammatory endotype is characterized by biomarkers that are downstream reflections of sustained inflammation in connective tissues (including C3M and CRPM) [2], which may in part explain the consistent association of IL-1ra with this endotype. In light of these results, IL-1ra may represent a therapeutic target and/or a modifiable biomarker for KOA subgroups driven by an inflammatory endotype.

Molecular endotyping holds the potential for targeted and personalized drug development in OA [1]. Before molecular endotyping can be used as a clinically feasible tool to enhance trial recruitment, endotypic assignments need to be stable and reliable on a patient-to-patient level within the length of a clinical trial [4, 6]. In this study, we found that the within-subject stability of the 15 cytokines were generally lower over approximately 18 months than for the 19 tissue turnover biomarkers used to describe the endotype subgroups [3, 4]. Most of the cytokines exhibited moderate-to-good within-participant stability over time whereas a majority of the tissue turnover biomarkers yielded good-to-excellent within-participant stability. While the majority of the included cytokines are important mediators of disease in OA, they did not exhibit consistent expression patterns over time for the endotype subgroups. Even for the subset of OA participants with a longitudinally stable endotype over 18 months (defined as being assigned to the same endotype over all three visits independently), cytokine fluctuations were observed to the same extent as the full population. Therefore, the results of this study highlight the importance and applicability of the choice of markers used for endotyping OA patients. The included cytokines may offer insight into the acute cytokine-load at the time of sampling but may not provide a stable assessment of the endotypic profile of OA patients.

Many of the tissue turnover biomarkers included in this work, such as C1M and C3M, are downstream reflections of the pathobiological processes, including inflammation, in the affected tissues and have been found to show consistent expression patterns over time for the three endotypes [4]. An independent clustering analysis based on the cytokine measurements alone did not provide meaningful cluster subgroups, as was observed with the tissue turnover biomarkers [3]. As a result, utilizing tissue turnover markers reflecting downstream consequences of cytokine-load rather than the cytokines themselves for molecular endotyping may offer more robust endotypic profiling of OA patients within the length of a clinical trial. However, while fluctuations are associated with the cytokines over time, such cytokine markers can be utilized pharmacodynamically or can provide valuable insight into the acute cytokine-load and activity of the disease.

Limitations of this study include the low number of cytokines considered in this study, caused by the limited number of cytokines quantified by the Luminex^®^ platform and the high percentage of missing data. 9/24 cytokines had missing data of > 50% at the first visit due to measurements being below the detection limit. As a result, cytokines of high relevance to the pathobiology of OA, such as IL-6, were excluded from this study. However, no endotype-specific patterns of missingness were observed for the cytokine panel. Reciprocal measurements of receptors of the cytokine panel, such as the IL-1 receptor, were not quantified and is a limitation of this study as they can influence the fluctuating levels of the cytokines included. Another limitation is that the cytokines were quantified in serum and not in or accompanied by synovial fluid. While analysis of serum represents a less invasive and potentially more clinically preferable sample type [21], it lacks the tissue specificity of that of synovial fluid. Limitation of this work also includes the low number of participants in this study (*n* = 277) and the lack of an independent cohort to evaluate the consistency of the findings, rendering this study exploratory in nature. Other clinical factors such as changes in comorbidities and use of medication could also influence the reported stability of the cytokine levels across visits and is a limitation of this work. Future work involves a more comprehensive quantification of cytokines, such as proteomic or transcriptomic profiling, to assess their association to the biomarker-based endotypes.

## Conclusions

This study performed longitudinal cytokine profiling of 15 pro- and anti-inflammatory cytokines to further the molecular understanding of the KOA endotypes driven by structural damage, low tissue turnover, and inflammation. This study found that the majority of the cytokines exhibited considerable fluctuations over time with no endotype-specific cytokine patterns in serum. As the only cytokine, IL-1ra was consistently elevated in the inflammatory endotype. While the cytokines included in this study offer insight into the acute cytokine-load of the disease, this study indicates that they may not be stable reflections of the endotypic profile of KOA patients over time compared to biochemical markers of tissue turnover. More research is needed on larger panels of cytokines to further the understanding of the pathology driving the KOA endotypes.

## Supplementary Information

Supplementary Material 1.

Supplementary Material 2.

## Data Availability

Data from IMI-APPROACH can be obtained upon reasonable request to the IMI-APPROACH Steering Committee.

## Abbreviations

ARGS-aggrecan: N-terminal neoepitope of aggrecanase-mediated degradation of aggrecan
BMI: Body mass index
C1M: Matrix metalloproteinase-mediated degradation fragment of type I collagen
C2M: Matrix metalloproteinase-mediated degradation fragment of type II collagen
C3M: Matrix metalloproteinase-mediated degradation fragment of type III collagen
C10C: C-terminal epitope of type X collagen
CI: Confidence interval
Coll2-1: N-terminal epitope on type II collagen degradation fragment
Coll2-1NO_2_: Nitrated form of Coll2-1
COMP: Cartilage oligomeric matrix protein
CRPM: Matrix metalloproteinase-mediated degradation fragment of C-reactive protein
GM-CSF: Granulocyte-macrophage colony-stimulating factor
HA: Hyaluronic acid
hsCRP: High-sensitivity C-reactive protein
ICC: Intraclass correlation coefficient
IFN: Interferon
IL: Interleukin
IL-1ra: Interleukin-1 receptor antagonist
IP: Interferon-γ-induced protein
IQR: Interquartile range
KOA: Knee osteoarthritis
IMI-APPROACH: Innovative Medicines Initiative Applied Public-Private Research enabling Osteoarthritis Clinical Headway
LMM: Linear mixed-effects model
MCP: Monocyte chemoattractant protein
MIP: Macrophage inflammatory protein
N-MID: N-terminal middle fragment of osteocalcin
OA: Osteoarthritis
PRO-C1: N-terminal propeptide of type I collagen
PRO-C2: Type IIB N-terminal propeptide of type II collagen
PRO-C4: N-terminal propeptide of type IV collagen
TNF: Tumor necrosis factor
sCTX-I: Serum C-terminal cross-linked, β-isomerized telopeptide of type I collagen
u-αCTX-I: Urinary C-terminal cross-linked, α-isomerized telopeptide of type I collagen
uCTX-II: Urinary C-terminal cross-linked telopeptide of type II collagen
VICM: Citrullinated vimentin
WOMAC: Western Ontario and McMaster Universities Arthritis Index
