# Supplementary material for: Cytokine profiling of molecular endotypes of knee osteoarthritis: insights from the IMI-APPROACH cohort
Source: Arthritis Res Ther. 2026 Jan 27;28:52. doi: 10.1186/s13075-026-03742-9 (PMC12918155; doi:10.1186/s13075-026-03742-9)
Supplement: Supplementary file 2 — Supplementary Material 2. [file 13075_2026_3742_MOESM2_ESM.docx]

**Supplementary Table 1.** Number of missing values due to being below lower limit of detection for 24 cytokines.

|  | **No. of missing values** | | |
| --- | --- | --- | --- |
| **Cytokine** | **Month 6** | **Month 12** | **Month 24** |
| CD40L | 242 (87%) | 195 (75%) | 188 (80%) |
| GM-CSF | 3 (1%) | 0 (0%) | 0 (0%) |
| Granzyme B | 274 (99%) | 257 (98%) | 231 (99%) |
| IFN-α | 237 (86%) | 147 (56%) | 206 (88%) |
| IFN-γ | 278 (100%) | 261 (100%) | 234 (100%) |
| IL-1α | 54 (19%) | 18 (7%) | 21 (9%) |
| IL-1β | 97 (35%) | 61 (23%) | 122 (52%) |
| IL-1ra | 0 (0%) | 0 (0%) | 1 (0%) |
| IL-2 | 90 (32%) | 41 (16%) | 67 (29%) |
| IL-4 | 255 (92%) | 203 (78%) | 222 (95%) |
| IL-6 | 275 (99%) | 258 (99%) | 231 (99%) |
| IL-8 | 0 (0%) | 0 (0%) | 0 (0%) |
| IL-10 | 21 (8%) | 3 (1%) | 11 (5%) |
| IL-12p70 | 97 (35%) | 41 (16%) | 108 (46%) |
| IL-13 | 244 (88%) | 203 (78%) | 196 (84%) |
| IL-15 | 60 (22%) | 13 (5%) | 28 (12%) |
| IL-17A | 2 (1%) | 21 (8%) | 29 (12%) |
| IL-33 | 255 (92%) | 225 (86%) | 191 (82%) |
| IP-10 | 0 (0%) | 0 (0%) | 0 (0%) |
| MCP-1 | 0 (0%) | 0 (0%) | 0 (0%) |
| MIP-1α | 139 (50%) | 63 (24%) | 129 (55%) |
| MIP-1β | 26 (9%) | 3 (1%) | 5 (2%) |
| PD-L1 | 242 (87%) | 216 (83%) | 194 (83%) |
| TNF-α | 9 (3%) | 3 (1%) | 16 (7%) |

Cytokines with > 50% missing values at month six were excluded from the study.

**Supplementary Table 2.** Percent change in geometric mean cytokine concentrations between endotypes estimated with linear-mixed effects models.

| **Visit** | **Cytokine** | **Reference** | **Contrast** | **Adj. for age, sex, BMI** | **Percent change**  **(95% CI)** | ***p*-value** | **Adj. *p*-value** |
| --- | --- | --- | --- | --- | --- | --- | --- |
| M006 | GM-CSF | Structural damage | Inflammatory | Yes No | 7.14 (-4.88, 20.68) 8.22 (-3.54, 21.53) | 0.261 0.182 | 0.766 0.544 |
| M006 | GM-CSF | Structural damage | Low turnover | Yes No | -8.06 (-18.29, 3.56) -7.60 (-17.88, 3.77) | 0.172 0.185 | 0.724 0.544 |
| M012 | GM-CSF | Structural damage | Inflammatory | Yes No | 5.34 (-6.95, 19.24) 6.72 (-5.64, 20.68) | 0.417 0.305 | 0.893 0.661 |
| M012 | GM-CSF | Structural damage | Low turnover | Yes No | -7.96 (-17.96, 3.36) -7.32 (-17.30, 3.98) | 0.165 0.198 | 0.724 0.552 |
| M024 | GM-CSF | Structural damage | Inflammatory | Yes No | 8.98 (-4.11, 23.74) 11.18 (-1.98, 25.99) | 0.189 0.101 | 0.724 0.383 |
| M024 | GM-CSF | Structural damage | Low turnover | Yes No | -8.88 (-18.94, 2.53) -9.06 (-19.10, 2.33) | 0.126 0.115 | 0.611 0.396 |
| M006 | GM-CSF | Inflammatory | Low turnover | Yes No | -14.10 (-23.97, -3.05) -14.70 (-24.50, -3.63) | 0.015 0.011 | 0.362 0.109 |
| M012 | GM-CSF | Inflammatory | Low turnover | Yes No | -12.63 (-23.36, -0.30) -13.06 (-23.74, -0.90) | 0.047 0.037 | 0.440 0.251 |
| M024 | GM-CSF | Inflammatory | Low turnover | Yes No | -16.39 (-26.66, -4.59) -18.13 (-28.18, -6.76) | 0.009 0.003 | 0.345 0.055 |
| M006 | IL-1α | Structural damage | Inflammatory | Yes No | -2.57 (-17.22, 14.68) -2.57 (-16.81, 14.22) | 0.756 0.751 | 0.976 0.932 |
| M006 | IL-1α | Structural damage | Low turnover | Yes No | -4.40 (-19.18, 13.09) -4.69 (-19.18, 12.41) | 0.603 0.571 | 0.944 0.859 |
| M012 | IL-1α | Structural damage | Inflammatory | Yes No | -2.76 (-17.14, 14.11) -1.59 (-16.05, 15.37) | 0.731 0.842 | 0.976 0.932 |
| M012 | IL-1α | Structural damage | Low turnover | Yes No | -2.37 (-16.31, 14.00) -0.20 (-14.36, 16.30) | 0.765 0.980 | 0.976 0.988 |
| M024 | IL-1α | Structural damage | Inflammatory | Yes No | 1.71 (-14.62, 20.92) 1.92 (-14.02, 20.92) | 0.850 0.827 | 0.979 0.932 |
| M024 | IL-1α | Structural damage | Low turnover | Yes No | -14.62 (-27.24, 0.10) -14.53 (-27.09, 0.20) | 0.055 0.055 | 0.474 0.298 |
| M006 | IL-1α | Inflammatory | Low turnover | Yes No | -1.88 (-17.22, 16.42) -2.18 (-17.63, 16.07) | 0.829 0.799 | 0.976 0.932 |
| M012 | IL-1α | Inflammatory | Low turnover | Yes No | 0.50 (-15.63, 19.60) 1.41 (-14.79, 20.68) | 0.959 0.873 | 0.984 0.949 |
| M024 | IL-1α | Inflammatory | Low turnover | Yes No | -16.05 (-29.53, 0.10) -16.14 (-29.60, -0.20) | 0.051 0.047 | 0.463 0.264 |
| M006 | IL-1β | Structural damage | Inflammatory | Yes No | 2.84 (-6.57, 13.09) 1.71 (-7.41, 11.63) | 0.577 0.725 | 0.944 0.932 |
| M006 | IL-1β | Structural damage | Low turnover | Yes No | 2.74 (-6.67, 13.09) 2.12 (-7.13, 12.30) | 0.582 0.662 | 0.944 0.918 |
| M012 | IL-1β | Structural damage | Inflammatory | Yes No | -2.96 (-11.49, 6.50) -0.70 (-9.43, 8.98) | 0.536 0.890 | 0.925 0.962 |
| M012 | IL-1β | Structural damage | Low turnover | Yes No | 3.56 (-5.16, 13.09) 4.60 (-4.30, 14.11) | 0.441 0.325 | 0.893 0.670 |
| M024 | IL-1β | Structural damage | Inflammatory | Yes No | 8.44 (-4.30, 22.88) 8.65 (-3.92, 22.75) | 0.210 0.189 | 0.724 0.545 |
| M024 | IL-1β | Structural damage | Low turnover | Yes No | 5.65 (-5.64, 18.53) 5.87 (-5.54, 18.65) | 0.343 0.325 | 0.850 0.670 |
| M006 | IL-1β | Inflammatory | Low turnover | Yes No | 0.00 (-9.70, 10.63) 0.40 (-9.34, 11.29) | 0.995 0.934 | 0.995 0.988 |
| M012 | IL-1β | Inflammatory | Low turnover | Yes No | 6.72 (-3.44, 17.82) 5.23 (-4.78, 16.30) | 0.210 0.321 | 0.724 0.670 |
| M024 | IL-1β | Inflammatory | Low turnover | Yes No | -2.57 (-14.36, 10.96) -2.57 (-14.19, 10.74) | 0.697 0.694 | 0.973 0.923 |
| M006 | IL-1ra | Structural damage | Inflammatory | Yes No | 22.14 (7.90, 38.40) 26.74 (11.96, 43.48) | 0.002  < 0.001 | 0.083  < 0.001 |
| M006 | IL-1ra | Structural damage | Low turnover | Yes No | 9.53 (-3.05, 23.61) 13.09 (0.10, 27.63) | 0.149 0.049 | 0.676 0.273 |
| **M012** | **IL-1ra** | **Structural damage** | **Inflammatory** | **Yes No** | **35.53 (19.01, 54.34) 39.38 (22.26, 58.88)** | **< 0.001  < 0.001** | **< 0.001  < 0.001** |
| M012 | IL-1ra | Structural damage | Low turnover | Yes No | 13.09 (0.40, 27.51) 15.37 (2.33, 30.08) | 0.045 0.020 | 0.440 0.172 |
| **M024** | **IL-1ra** | **Structural damage** | **Inflammatory** | **Yes No** | **39.65 (22.26, 59.36) 46.08 (28.02, 66.70)** | **< 0.001  < 0.001** | **< 0.001  < 0.001** |
| M024 | IL-1ra | Structural damage | Low turnover | Yes No | 10.19 (-2.37, 24.48) 13.66 (0.60, 28.27) | 0.120 0.042 | 0.611 0.251 |
| M006 | IL-1ra | Inflammatory | Low turnover | Yes No | -10.42 (-21.02, 1.61) -10.86 (-21.49, 1.31) | 0.090 0.079 | 0.611 0.335 |
| M012 | IL-1ra | Inflammatory | Low turnover | Yes No | -16.47 (-27.17, -4.30) -17.22 (-27.89, -5.07) | 0.010 0.007 | 0.362 0.084 |
| **M024** | **IL-1ra** | **Inflammatory** | **Low turnover** | **Yes No** | **-21.02 (-31.13, -9.52) -22.20 (-32.16, -10.77)** | **0.001  < 0.001** | **0.047  < 0.001** |
| M006 | IL-2 | Structural damage | Inflammatory | Yes No | -3.15 (-13.67, 8.65) -3.34 (-13.58, 8.11) | 0.591 0.555 | 0.944 0.858 |
| M006 | IL-2 | Structural damage | Low turnover | Yes No | 3.05 (-8.24, 15.60) 2.33 (-8.70, 14.80) | 0.620 0.692 | 0.948 0.923 |
| M012 | IL-2 | Structural damage | Inflammatory | Yes No | -2.57 (-12.63, 8.55) -0.30 (-10.60, 11.18) | 0.640 0.956 | 0.948 0.988 |
| M012 | IL-2 | Structural damage | Low turnover | Yes No | -0.40 (-10.15, 10.41) 0.80 (-9.15, 11.74) | 0.934 0.885 | 0.979 0.959 |
| M024 | IL-2 | Structural damage | Inflammatory | Yes No | 13.31 (-0.10, 28.40) 10.74 (-2.08, 25.23) | 0.054 0.106 | 0.474 0.383 |
| M024 | IL-2 | Structural damage | Low turnover | Yes No | 5.44 (-6.01, 18.41) 4.08 (-7.23, 16.65) | 0.371 0.497 | 0.868 0.805 |
| M006 | IL-2 | Inflammatory | Low turnover | Yes No | 6.40 (-5.64, 19.84) 5.87 (-6.20, 19.48) | 0.319 0.357 | 0.819 0.705 |
| M012 | IL-2 | Inflammatory | Low turnover | Yes No | 2.22 (-9.15, 15.03) 1.11 (-10.24, 13.88) | 0.721 0.860 | 0.976 0.937 |
| M024 | IL-2 | Inflammatory | Low turnover | Yes No | -6.95 (-18.21, 5.97) -6.01 (-17.47, 6.93) | 0.286 0.350 | 0.774 0.701 |
| M006 | IL-8 | Structural damage | Inflammatory | Yes No | -2.96 (-10.60, 5.34)  -0.50 (-8.15, 7.68) | 0.476 0.894 | 0.896 0.965 |
| M006 | IL-8 | Structural damage | Low turnover | Yes No | -0.10 (-7.78, 8.33) 1.51 (-6.29, 9.86) | 0.990 0.719 | 0.991 0.932 |
| M012 | IL-8 | Structural damage | Inflammatory | Yes No | -5.45 (-13.15, 3.05) -3.44 (-11.22, 5.02) | 0.204 0.420 | 0.724 0.754 |
| M012 | IL-8 | Structural damage | Low turnover | Yes No | 1.11 (-6.57, 9.42) 1.51 (-6.11, 9.75) | 0.785 0.707 | 0.976 0.923 |
| M024 | IL-8 | Structural damage | Inflammatory | Yes No | 8.33 (-0.70, 18.18) 8.98 (0.00, 18.65) | 0.073 0.052 | 0.551 0.284 |
| M024 | IL-8 | Structural damage | Low turnover | Yes No | 2.02 (-5.82, 10.41) 1.82 (-5.92, 10.30) | 0.632 0.655 | 0.948 0.918 |
| M006 | IL-8 | Inflammatory | Low turnover | Yes No | 3.05 (-5.16, 11.85) 2.02 (-6.11, 10.85) | 0.489 0.636 | 0.896 0.904 |
| M012 | IL-8 | Inflammatory | Low turnover | Yes No | 6.93 (-2.27, 17.00) 5.13 (-3.92, 15.03) | 0.149 0.277 | 0.676 0.621 |
| M024 | IL-8 | Inflammatory | Low turnover | Yes No | -5.92 (-14.02, 2.94) -6.48 (-14.53, 2.22) | 0.190 0.142 | 0.724 0.459 |
| M006 | IL-10 | Structural damage | Inflammatory | Yes No | 24.61 (3.77, 49.63) 28.79 (7.79, 53.88) | 0.020 0.006 | 0.362 0.084 |
| M006 | IL-10 | Structural damage | Low turnover | Yes No | 1.11 (-15.80, 21.53) 2.84 (-14.19, 23.12) | 0.903 0.765 | 0.979 0.932 |
| M012 | IL-10 | Structural damage | Inflammatory | Yes No | 7.36 (-10.86, 29.30) 8.87 (-9.43, 31.00) | 0.457 0.367 | 0.893 0.706 |
| M012 | IL-10 | Structural damage | Low turnover | Yes No | -9.52 (-24.04, 7.79) -9.24 (-23.74, 8.00) | 0.267 0.278 | 0.767 0.621 |
| M024 | IL-10 | Structural damage | Inflammatory | Yes No | 17.47 (-3.25, 42.62) 22.38 (1.11, 48.14) | 0.106 0.039 | 0.611 0.251 |
| M024 | IL-10 | Structural damage | Low turnover | Yes No | 3.77 (-13.58, 24.61) 3.15 (-14.10, 23.86) | 0.692 0.744 | 0.973 0.932 |
| M006 | IL-10 | Inflammatory | Low turnover | Yes No | -18.86 (-32.70, -2.08) -20.15 (-33.83, -3.63) | 0.031 0.020 | 0.396 0.172 |
| M012 | IL-10 | Inflammatory | Low turnover | Yes No | -15.72 (-30.79, 2.63) -16.64 (-31.55, 1.51) | 0.093 0.072 | 0.611 0.324 |
| M024 | IL-10 | Inflammatory | Low turnover | Yes No | -11.57 (-27.89, 8.33) -15.72 (-31.13, 3.15) | 0.238 0.099 | 0.755 0.383 |
| M006 | IL-12p70 | Structural damage | Inflammatory | Yes No | -10.95 (-26.66, 8.22) -8.61 (-24.04, 9.97) | 0.251 0.346 | 0.764 0.700 |
| M006 | IL-12p70 | Structural damage | Low turnover | Yes No | -2.86 (-20.63, 18.89) -0.20 (-18.13, 21.53) | 0.782 0.981 | 0.976 0.988 |
| M012 | IL-12p70 | Structural damage | Inflammatory | Yes No | 3.36 (-14.02, 24.11) 3.05 (-14.02, 23.61) | 0.731 0.744 | 0.976 0.932 |
| M012 | IL-12p70 | Structural damage | Low turnover | Yes No | -1.00 (-16.22, 17.00) 0.10 (-15.30, 18.18) | 0.909 0.994 | 0.979 0.996 |
| M024 | IL-12p70 | Structural damage | Inflammatory | Yes No | -3.34 (-23.74, 22.51) -9.52 (-28.32, 14.22) | 0.780 0.405 | 0.976 0.743 |
| M024 | IL-12p70 | Structural damage | Low turnover | Yes No | 5.97 (-14.96, 32.18) 3.05 (-17.14, 28.27) | 0.609 0.787 | 0.948 0.932 |
| M006 | IL-12p70 | Inflammatory | Low turnover | Yes No | 9.09 (-10.95, 33.51) 9.09 (-10.86, 33.64) | 0.407 0.402 | 0.892 0.741 |
| M012 | IL-12p70 | Inflammatory | Low turnover | Yes No | -4.21 (-20.78, 15.95) -2.96 (-19.75, 17.35) | 0.666 0.760 | 0.961 0.932 |
| M024 | IL-12p70 | Inflammatory | Low turnover | Yes No | 9.64 (-13.06, 38.40) 13.88 (-9.61, 43.48) | 0.442 0.273 | 0.893 0.621 |
| M006 | IL-15 | Structural damage | Inflammatory | Yes No | 6.18 ( -1.49, 14.34) 5.55 ( -1.88, 13.66) | 0.121 0.150 | 0.611 0.469 |
| M006 | IL-15 | Structural damage | Low turnover | Yes No | 0.40 (-6.85, 8.22)  0.00 (-7.13, 7.68) | 0.924 0.999 | 0.979 0.999 |
| M012 | IL-15 | Structural damage | Inflammatory | Yes No | -1.78 (-8.61, 5.55) -0.70 (-7.60, 6.72) | 0.629 0.845 | 0.948 0.932 |
| M012 | IL-15 | Structural damage | Low turnover | Yes No | -2.47 (-8.88, 4.39) -1.69 (-8.15, 5.13) | 0.479 0.615 | 0.896 0.890 |
| M024 | IL-15 | Structural damage | Inflammatory | Yes No | 3.36 (-4.40, 11.85) 2.74 (-4.88, 11.07) | 0.407 0.486 | 0.892 0.797 |
| M024 | IL-15 | Structural damage | Low turnover | Yes No | 4.39 (-2.96, 12.30) 3.77 (-3.44, 11.63) | 0.255 0.317 | 0.764 0.670 |
| M006 | IL-15 | Inflammatory | Low turnover | Yes No | -5.45 (-12.63, 2.33) -5.26 (-12.54, 2.53) | 0.169 0.184 | 0.724 0.544 |
| M012 | IL-15 | Inflammatory | Low turnover | Yes No | -0.70 (-8.15, 7.47) -1.00 (-8.52, 7.04) | 0.865 0.799 | 0.979 0.932 |
| M024 | IL-15 | Inflammatory | Low turnover | Yes No | 0.90 (-6.95, 9.53) 1.01 (-6.95, 9.53) | 0.825 0.811 | 0.976 0.932 |
| M006 | IL-17A | Structural damage | Inflammatory | Yes No | 0.90 (-9.61, 12.75) 0.60 (-9.61, 12.08) | 0.867 0.907 | 0.979 0.977 |
| M006 | IL-17A | Structural damage | Low turnover | Yes No | 3.56 (-7.41, 15.72) 3.67 (-7.23, 15.72) | 0.546 0.529 | 0.936 0.839 |
| M012 | IL-17A | Structural damage | Inflammatory | Yes No | -1.98 (-12.89, 10.19) 0.40 (-10.68, 12.98) | 0.737 0.943 | 0.976 0.988 |
| M012 | IL-17A | Structural damage | Low turnover | Yes No | -0.40 (-11.22, 11.74) 1.51 (-9.61, 13.88) | 0.948 0.804 | 0.980 0.932 |
| M024 | IL-17A | Structural damage | Inflammatory | Yes No | 2.63 (-9.79, 16.65) 2.63 (-9.52, 16.42) | 0.694 0.689 | 0.973 0.923 |
| M024 | IL-17A | Structural damage | Low turnover | Yes No | -5.73 (-16.64, 6.72)  -5.26 (-16.31, 7.25) | 0.356 0.394 | 0.859 0.735 |
| M006 | IL-17A | Inflammatory | Low turnover | Yes No | 2.53 (-8.42, 14.80) 2.94 (-8.15, 15.37) | 0.666 0.616 | 0.961 0.890 |
| M012 | IL-17A | Inflammatory | Low turnover | Yes No | 1.71 (-10.51, 15.49) 1.01 (-11.13, 14.91) | 0.801 0.874 | 0.976 0.949 |
| M024 | IL-17A | Inflammatory | Low turnover | Yes No | -8.06 (-19.59, 5.13) -7.69 (-19.27, 5.44) | 0.218 0.240 | 0.724 0.578 |
| M006 | IP-10 | Structural damage | Inflammatory | Yes No | 3.15 (-6.48, 13.77) 6.29 (-3.34, 17.00) | 0.541 0.212 | 0.932 0.569 |
| M006 | IP-10 | Structural damage | Low turnover | Yes No | -9.70 (-17.96, -0.60) -8.24 (-16.56, 0.90) | 0.039 0.077 | 0.412 0.333 |
| M012 | IP-10 | Structural damage | Inflammatory | Yes No | 3.77 (-6.29, 14.91) 4.81 (-5.26, 16.07) | 0.479 0.360 | 0.896 0.705 |
| M012 | IP-10 | Structural damage | Low turnover | Yes No | -5.64 (-14.10, 3.77) -5.82 (-14.19, 3.46) | 0.235 0.212 | 0.755 0.569 |
| M024 | IP-10 | Structural damage | Inflammatory | Yes No | 3.98 (-6.29, 15.37) 6.18 (-4.21, 17.59) | 0.462 0.257 | 0.893 0.606 |
| M024 | IP-10 | Structural damage | Low turnover | Yes No | -4.02 (-12.80, 5.65)  -4.21 (-12.89, 5.34) | 0.406 0.379 | 0.892 0.719 |
| M006 | IP-10 | Inflammatory | Low turnover | Yes No | -12.45 (-20.71, -3.34) -13.76 (-21.89, -4.69) | 0.009 0.004 | 0.345 0.067 |
| M012 | IP-10 | Inflammatory | Low turnover | Yes No | -9.06 (-18.29, 1.31) -10.15 (-19.35, 0.00) | 0.087 0.051 | 0.611 0.281 |
| M024 | IP-10 | Inflammatory | Low turnover | Yes No | -7.69 (-17.14, 2.74) -9.79 (-18.86, 0.40) | 0.147 0.062 | 0.673 0.302 |
| M006 | MCP-1 | Structural damage | Inflammatory | Yes No | 2.63 (-4.11, 9.86) 4.29 (-2.37, 11.40) | 0.453 0.218 | 0.893 0.569 |
| M006 | MCP-1 | Structural damage | Low turnover | Yes No | -2.27 (-8.52, 4.29)  -1.29 (-7.50, 5.44) | 0.489 0.703 | 0.896 0.923 |
| M012 | MCP-1 | Structural damage | Inflammatory | Yes No | 3.05 (-4.02, 10.63) 5.34 (-1.78, 12.98) | 0.408 0.150 | 0.893 0.469 |
| M012 | MCP-1 | Structural damage | Low turnover | Yes No | 5.44 (-1.19, 12.41) 6.29 (-0.30, 13.31) | 0.112 0.066 | 0.611 0.319 |
| **M024** | **MCP-1** | **Structural damage** | **Inflammatory** | **Yes No** | **13.31 (5.44, 21.65) 13.54 (5.87, 21.90)** | **0.001  < 0.001** | **0.047  < 0.001** |
| M024 | MCP-1 | Structural damage | Low turnover | Yes No | 5.65 (-1.00, 12.64) 5.76 (-0.80, 12.86) | 0.103 0.091 | 0.611 0.372 |
| M006 | MCP-1 | Inflammatory | Low turnover | Yes No | -4.88 (-11.13, 1.92) -5.35 (-11.57, 1.41) | 0.158 0.120 | 0.714 0.411 |
| M012 | MCP-1 | Inflammatory | Low turnover | Yes No | 2.33 (-4.97, 10.19)  0.90 (-6.29, 8.65) | 0.551 0.813 | 0.940 0.932 |
| M024 | MCP-1 | Inflammatory | Low turnover | Yes No | -6.76 (-13.41, 0.40) -6.85 (-13.41, 0.30) | 0.065 0.060 | 0.526 0.302 |
| M006 | MIP-1α | Structural damage | Inflammatory | Yes No | -8.24 (-23.59, 10.19) -8.79 (-23.51, 8.76) | 0.362 0.305 | 0.859 0.661 |
| M006 | MIP-1α | Structural damage | Low turnover | Yes No | -6.39 (-20.63, 10.52) -7.23 (-21.02, 9.09) | 0.442 0.363 | 0.893 0.705 |
| M012 | MIP-1α | Structural damage | Inflammatory | Yes No | -2.86 (-17.14, 13.77) -3.92 (-17.80, 12.19) | 0.722 0.616 | 0.976 0.890 |
| M012 | MIP-1α | Structural damage | Low turnover | Yes No | 1.61 (-11.22, 16.30) 0.50 (-12.10, 15.03) | 0.819 0.938 | 0.976 0.988 |
| M024 | MIP-1α | Structural damage | Inflammatory | Yes No | 22.75 (-0.50, 51.29) 23.12 (1.11, 49.93) | 0.060 0.041 | 0.505 0.251 |
| M024 | MIP-1α | Structural damage | Low turnover | Yes No | 20.68 (0.60, 44.77) 20.08 (0.50, 43.62) | 0.046 0.047 | 0.440 0.264 |
| M006 | MIP-1α | Inflammatory | Low turnover | Yes No | 2.12 (-14.53, 21.90) 1.71 (-14.79, 21.53) | 0.822 0.849 | 0.976 0.934 |
| M012 | MIP-1α | Inflammatory | Low turnover | Yes No | 4.60 (-11.49, 23.61) 4.60 (-11.22, 23.37) | 0.601 0.592 | 0.944 0.879 |
| M024 | MIP-1α | Inflammatory | Low turnover | Yes No | -1.59 (-20.47, 21.77) -2.37 (-20.71, 20.20) | 0.884 0.821 | 0.979 0.932 |
| M006 | MIP-1β | Structural damage | Inflammatory | Yes No | 6.93 (-3.05, 18.06) 8.00 (-1.98, 18.89) | 0.182 0.123 | 0.724 0.418 |
| M006 | MIP-1β | Structural damage | Low turnover | Yes No | 3.25 (-6.29, 13.77) 3.87 (-5.73, 14.45) | 0.523 0.449 | 0.911 0.783 |
| M012 | MIP-1β | Structural damage | Inflammatory | Yes No | 4.39 (-5.54, 15.37) 4.39 (-5.54, 15.37) | 0.398 0.403 | 0.890 0.741 |
| M012 | MIP-1β | Structural damage | Low turnover | Yes No | 5.13 (-4.21, 15.37) 5.76 (-3.63, 16.07) | 0.292 0.241 | 0.781 0.578 |
| M024 | MIP-1β | Structural damage | Inflammatory | Yes No | 12.64 (1.61, 24.98) 12.41 (1.41, 24.61) | 0.024 0.026 | 0.388 0.203 |
| M024 | MIP-1β | Structural damage | Low turnover | Yes No | -0.30 (-9.34, 9.75) -0.10 (-9.24, 9.97) | 0.953 0.978 | 0.980 0.988 |
| M006 | MIP-1β | Inflammatory | Low turnover | Yes No | -3.44 (-12.80, 6.82) -3.82 (-13.24, 6.61) | 0.497 0.461 | 0.897 0.784 |
| M012 | MIP-1β | Inflammatory | Low turnover | Yes No | 0.70 (-9.43, 11.96) 1.31 (-8.97, 12.75) | 0.900 0.808 | 0.979 0.932 |
| M024 | MIP-1β | Inflammatory | Low turnover | Yes No | -11.49 (-20.39, -1.59) -11.13 (-20.15, -1.19) | 0.025 0.030 | 0.393 0.219 |
| M006 | TNF-α | Structural damage | Inflammatory | Yes No | 1.71 ( -9.52, 14.45) 0.20 (-10.60, 12.30) | 0.776 0.975 | 0.976 0.988 |
| M006 | TNF-α | Structural damage | Low turnover | Yes No | -3.73 (-14.44, 8.33) -4.59 (-15.13, 7.14) | 0.534 0.430 | 0.924 0.758 |
| M012 | TNF-α | Structural damage | Inflammatory | Yes No | 6.50 (-5.82, 20.56) 8.22 (-4.11, 22.26) | 0.314 0.204 | 0.816 0.560 |
| M012 | TNF-α | Structural damage | Low turnover | Yes No | -4.50 (-14.70, 7.04) -2.57 (-12.98, 9.20) | 0.436 0.657 | 0.893 0.918 |
| M024 | TNF-α | Structural damage | Inflammatory | Yes No | 15.26 (1.41, 31.00) 17.35 (3.46, 33.24) | 0.031 0.013 | 0.396 0.119 |
| M024 | TNF-α | Structural damage | Low turnover | Yes No | -1.39 (-12.45, 11.07) -1.19 (-12.28, 11.29) | 0.821 0.846 | 0.976 0.932 |
| M006 | TNF-α | Inflammatory | Low turnover | Yes No | -5.35 (-16.05, 6.72) -4.78 (-15.63, 7.36) | 0.374 0.428 | 0.868 0.758 |
| M012 | TNF-α | Inflammatory | Low turnover | Yes No | -10.33 (-21.18, 2.02) -9.97 (-20.86, 2.43) | 0.101 0.114 | 0.611 0.395 |
| M024 | TNF-α | Inflammatory | Low turnover | Yes No | -14.44 (-25.17, -2.18) -15.80 (-26.36, -3.92) | 0.023 0.011 | 0.378 0.109 |

Geometric means of percent changes between the three endotypes were estimated with linear-mixed effects models, adjusting for participant-specific random effects. Changes were estimated with and without adjusting for age, sex, and body mass index (BMI). P-values were Benjamini-Hochberg-adjusted for multiple comparisons. CI, confidence interval; M006, month six; M012, month 12; M024, month 24.

**Supplementary Table 3.** Changes in clinical characteristics of participants in lower and upper IL-1ra expression quartile within the inflammatory endotype.

|  | Low IL-1ra inflammatory endotype (*n* = 23)^a^ | High IL-1ra inflammatory endotype (*n* = 23)^b^ | *p*-value | Adjusted *p*-value |
| --- | --- | --- | --- | --- |
| ∆BMI (kg/m^2^)  Median (Q1, Q3)  Mean (SD) | -0.16 (-0.74, 0.55)  -0.16 (1.34) | -0.36 (-0.67, 0.42)  -0.03 (1.43) | 0.553 | 0.664 |
| ∆KL grade  0  1 | 18 (94.7%)  1 (5.3%) | 12 (92.3%)  1 (7.7%) | 1 | 1 |
| ∆JSW medial (mm)  Median (Q1, Q3)  Mean (SD) | -0.31 (-0.41, 0.17)  -0.21 (0.43) | 0 (-0.45, 0.54)  -0.01 (0.66) | 0.328 | 0.656 |
| ∆WOMAC Pain (%)  Median (Q1, Q3)  Mean (SD) | 0 (-15, 10)  -1.4 (19.6) | 5 (-11.2, 10)  0.9 (14.5) | 0.128 | 0.656 |
| ∆WOMAC Function (%)  Median (Q1, Q3)  Mean (SD) | -4.4 (-7.4, 5.9)  -2.9 (19.9) | -0.7 (-11.8, 4.8)  -2.3 (10.8) | 0.294 | 0.656 |
| ∆WOMAC Stiffness (%)  Median (Q1, Q3)  Mean (SD) | 0 (-12.5, 12.5)  -1.9 (21.2) | 0 (-12.5, 12.5)  -4.4 (20.2) | 0.499 | 0.664 |

Target knee data was considered. Changes were calculated between 24- and six-month visits. Higher Western Ontario and McMaster Universities Osteoarthritis Index (WOMAC) scores indicate worse symptoms. Pearson’s χ^2^ test was used for categorical variables and linear models adjusting for levels at the month six visit was used for numerical variables. P-values were Benjamini-Hochberg-adjusted for multiple comparisons. IL-1ra, interleukin-1 receptor antagonist; KL, Kellgren-Lawrence; JSW, joint-space width; SD, standard deviation.

^a^Missing data: BMI (*n* = 2), KL grade (*n* = 4), JSW medial (*n* = 4), WOMAC Pain (*n* = 2), WOMAC Function (*n* = 2), WOMAC Stiffness (*n* = 3).

^b^Missing data: BMI (*n* = 7), KL grade (*n* = 10), JSW medial (*n* = 9), WOMAC Pain (*n* = 7), WOMAC Function (*n* = 11), WOMAC Stiffness (*n* = 6).

**Supplementary Table 4.** Participants with a longitudinally stable endotype within the lower and upper IL-1ra expression quartile subgroups of the inflammatory endotype.

| **Longitudinal stability pattern** | **Low IL-1ra inflammatory endotype (*n* = 23)** | **High IL-1ra inflammatory endotype (*n* = 23)** |
| --- | --- | --- |
| Stable | 10 | 11 |
| Unstable | 10 | 6 |
| Unknown | 3 | 6 |

Longitudinal stability was defined as being assigned to the same endotype at each visit (month six, 12, and 24) independently [4]. Unknown longitudinal stability pattern refers to participants that either dropped or have a missing visit.


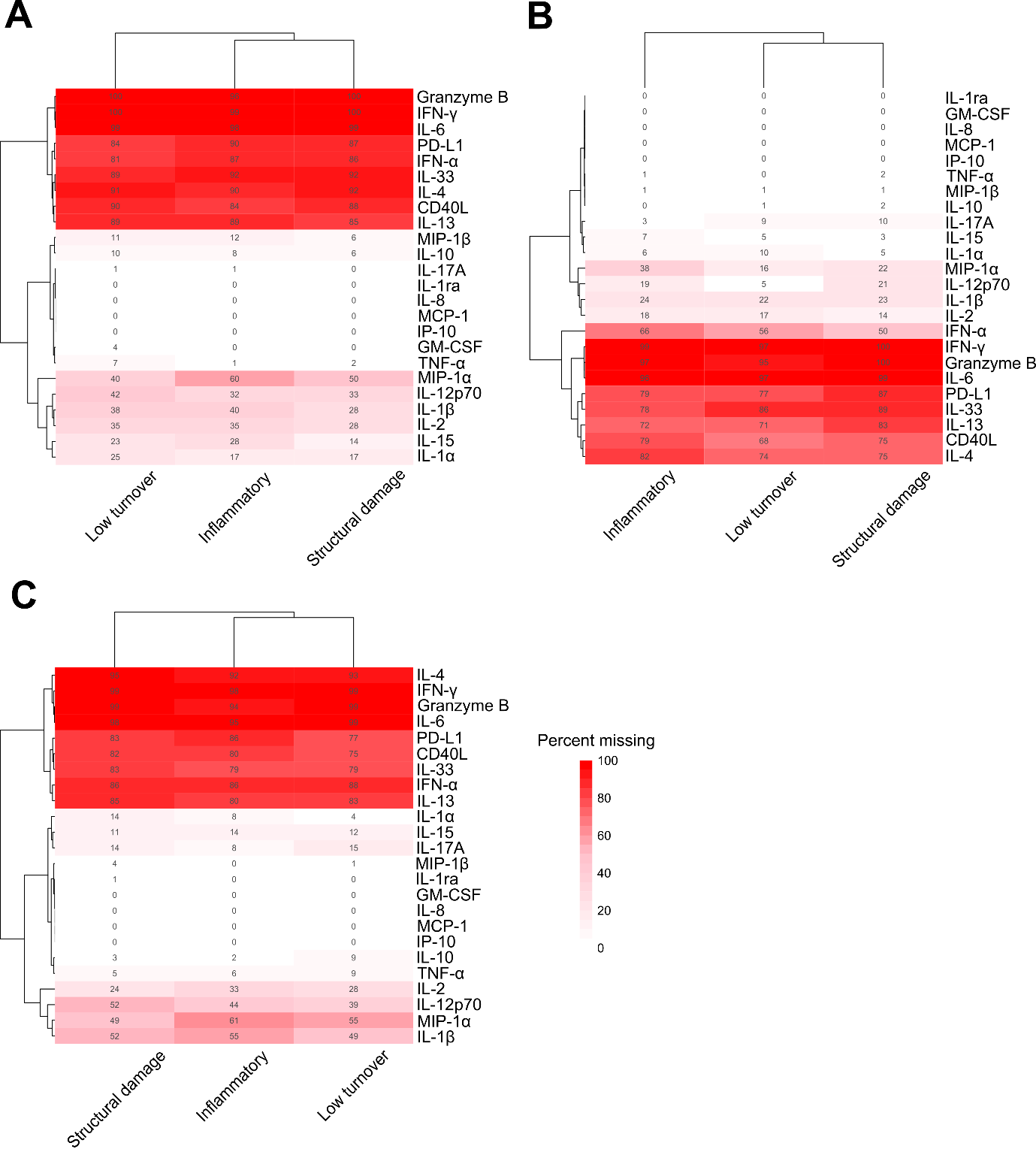


**Supplementary Fig. 1**. Percent missingness of cytokines for endotypes at A) six-, B) 12-, and C) 24-month visits.

**
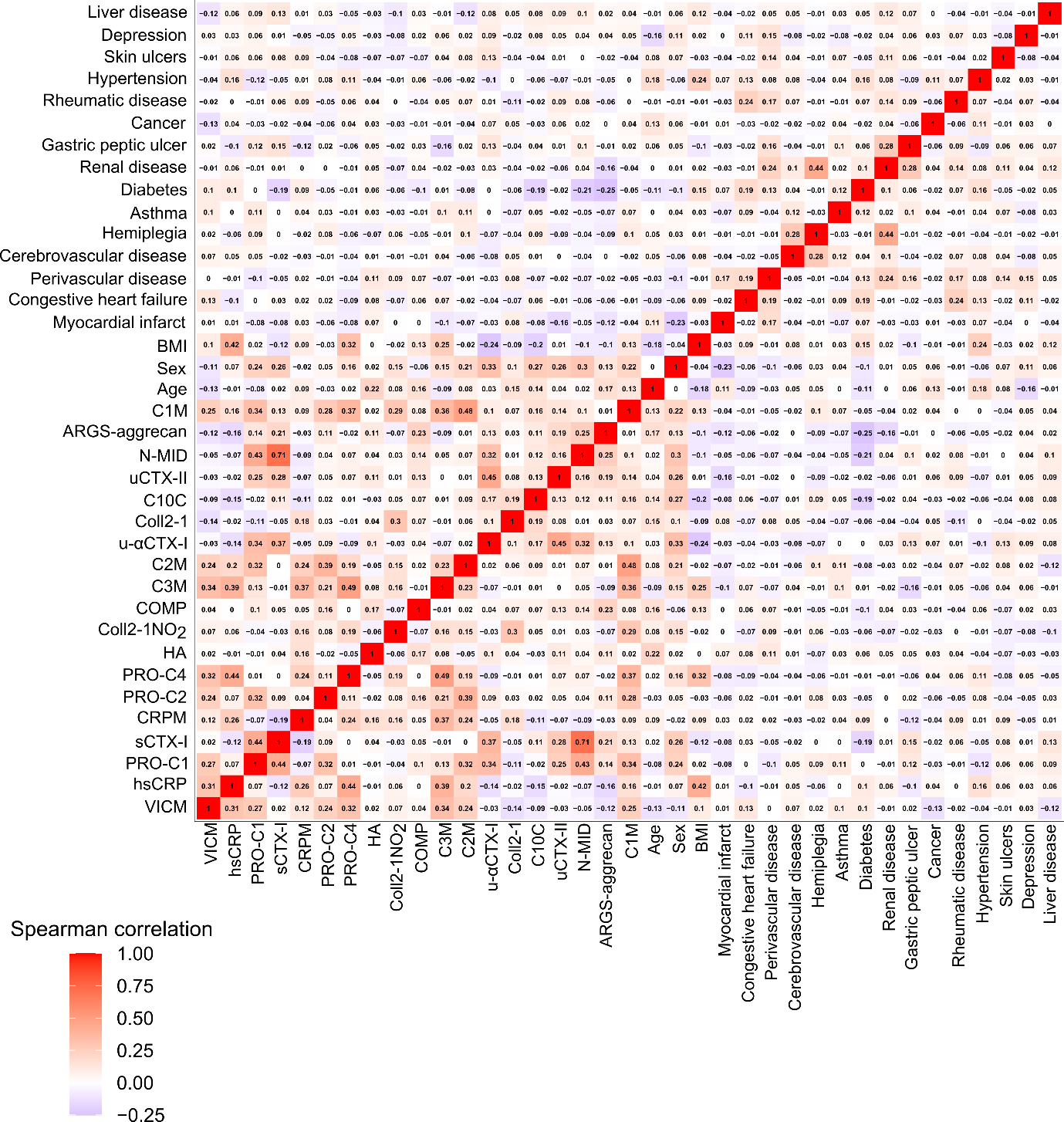
**

**Supplementary Fig. 2.** Spearman’s correlations between tissue-turnover biomarkers, clinical characteristics, reported comorbidities at the six-month visit.


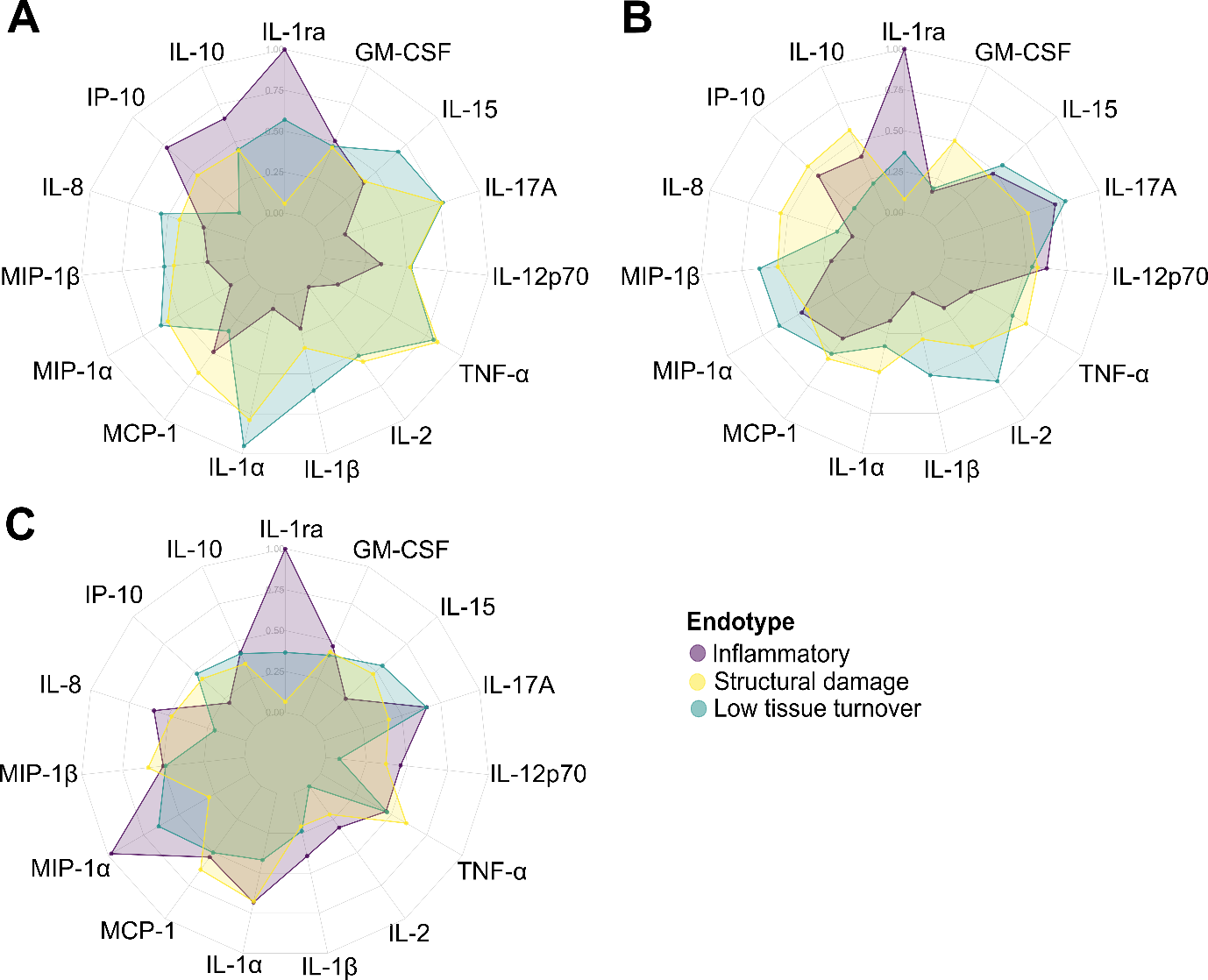


**Supplementary Fig. 3.** Cytokine profiles of longitudinally stable endotypes at the A) six-, B) 12-, and C) 24-month visits.


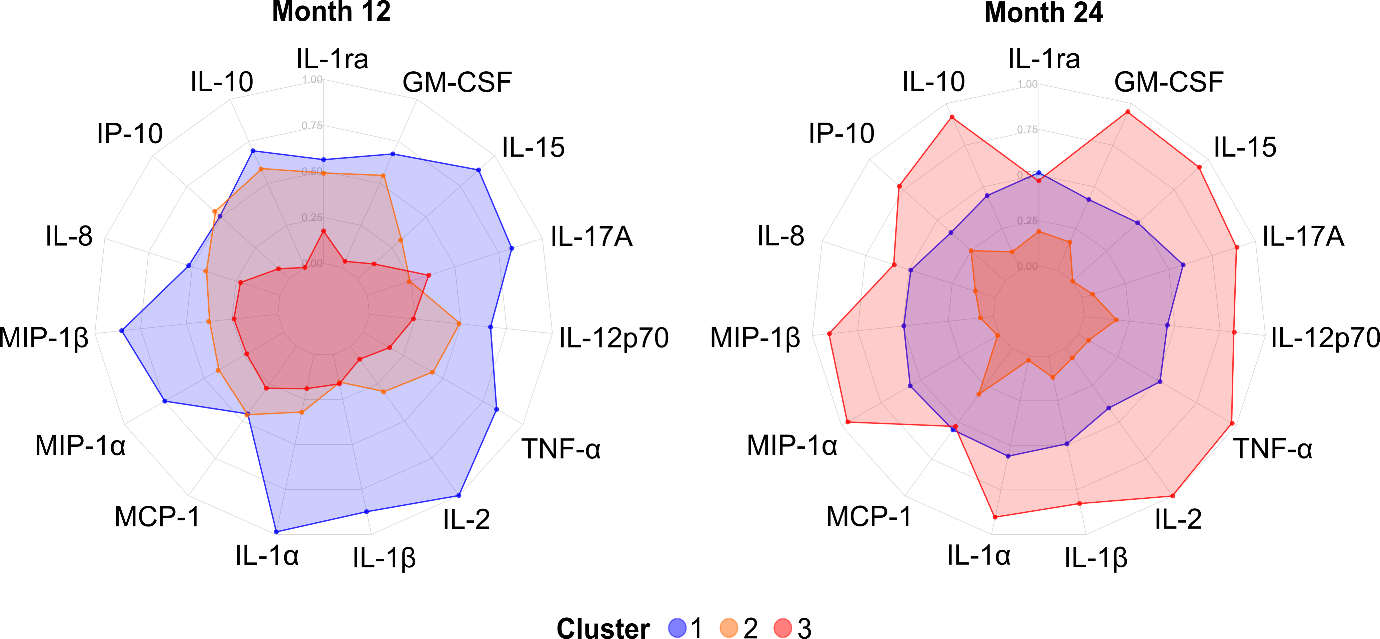


**Supplementary Fig. 4.** Cytokine profiles of three clusters obtained by *k*-means clustering of sex-specific z-score scaled cytokine levels for month 12 and 24.
